# The Dry-Weight Dilemma: Survey Results Based on a Qualitative Interview Study on Fluid Overload in Hemodialysis

**DOI:** 10.1016/j.xkme.2026.101320

**Published:** 2026-03-10

**Authors:** Vincent Rathkolb, Julian Krauß, Sebastian Mußnig, Simon Krenn, Christoph Matthias, Hannah Mayfurth, Viktoria Tinhof, Dragan Copic, Maximilian Waller, Matthias Lorenz, Sabine Schmaldienst, Ulrich Kropiunigg, David F. Keane, Manfred Hecking

**Affiliations:** 1 Clinical Division of Nephrology & Dialysis, Department of Internal Medicine III, Medical University of Vienna, Vienna, Austria; 2 Department of Epidemiology, Center for Public Health, Medical University of Vienna, Vienna, Austria; 3 Department of Medical Psychology, Medical University of Vienna, Vienna, Austria; 4 Clinical Division of Nephrology & Dialysis, Department of Internal Medicine I, Clinic Favoriten, Vienna, Austria; 5 AIT Austrian Institute of Technology GmbH, Center for Health & Bioresources, Medical Signal Analysis, Vienna, Austria; 6 Vienna Dialysis Center, Vienna, Austria; 7 CÚRAM Science Foudation Ireland Research Centre for Medical Devices, Health Research Board-Clinical Research Facility Galway, National University of Ireland Galway, Galway, Ireland

**Keywords:** Hemodialysis, Fluid overload, Dry weight, Bioimpedance spectroscopy, Fluid management, Patient perspectives, Patient engagement, Interdialytic weight gain

## Abstract

**Rationale & Objective:**

Chronic fluid overload (FO) can be detected using bioimpedance spectroscopy (BIS) and is associated with higher mortality risk than interdialytic weight gain in patients receiving hemodialysis (HD). This study aimed to explore patients’ perspectives and comprehension of FO and dry weight.

**Study Design:**

Qualitative study arm of an exploratory, nonrandomized observational study.

**Setting & Participants:**

Semistructured interviews were conducted in 25 HD patients at Vienna General Hospital. A survey was developed based on the interview data and distributed across 3 HD centers (n = 148).

**Analytical Approach:**

A mixed-methods approach was applied: grounded theory guided qualitative coding, whereas survey data were analyzed descriptively.

**Results:**

11 of 25 interviewed patients had chronic FO > 15% BIS-defined extracellular volume predialysis. Theme 1, “Being aware of fluid restrictions,” contained the following subthemes: (1a) “Restrictive fluid intake is the major burden,” (1b) “Symptom awareness of FO,” (1c) “Misconception of chronic FO,” (1d) “Unawareness of BIS,” and (1e) “neglected salt restriction.” Subthemes 1c and 1d were reinforced by the survey, in which 54.7% fully or partially agreed that chronic FO occurs between HD sessions (misunderstood as interdialytic weight gain). Theme 2, “Dry weight is a feel-good factor,” contained subthemes, (2a) “Inconclusive definitions of dry weight,” (2b) “Better too high than too low,” (2c) “Self-management of dry-weight prescription,” and (2d) “Uncertainties in weight documentation.” In our survey, 77.9% reported being able to define dry weight; 64.2% fully or partially agreed they currently had the correct dry weight; and 65.0% did not feel overhydrated (chronic FO was prevalent in ∼40%). 64.5% were not interested in participating in an educational event on dry weight and chronic FO.

**Limitations:**

Sample size, gender bias, and the survey was not validated externally.

**Conclusions:**

Substantial knowledge gaps existed regarding dry weight and chronic FO, necessitating improved patient engagement strategies into fluid management and further research into the underlying reasons for patients’ disinterest.

## Plain-Language Summary

Inadequate control of fluid status in hemodialysis patients is associated with increased cardiovascular risk and mortality. This study examined how patients understand chronic fluid overload (FO) and dry weight, 2 clinically important but often misunderstood concepts. Based on qualitative interviews and a follow-up survey, many patients confused chronic FO with short-term weight gain and were unfamiliar with diagnostic tools such as bioimpedance spectroscopy. Despite the high prevalence of FO, most patients believed their fluid status was adequate and showed limited interest in education. These findings highlight a disconnection between clinical risk and patient perception, emphasizing the need for targeted communication strategies to improve patient understanding, engagement, and adherence in fluid management.

Every patient receiving hemodialysis (HD), before and during each treatment session, is confronted with the decision of how much fluid should be removed.^1^ Complications of high fluid removal and the burden of fluid restriction make it challenging to maintain appropriate fluid management, in which hypovolemia and fluid overload (FO) must be kept in balance.^2^

An influential publication described interdialytic weight gain (IDWG) as fluid retention,^3^ although results from bioimpedance spectroscopy (BIS)^4^ made clear that chronic FO and IDWG are not one and the same.^5^ At least from 2009 onward,^6^ BIS-detected predialysis FO, defined as >15% extracellular water that corresponds to 2.5 L in a typical patient, was shown to be an independent risk factor for mortality.^7^, ^8^, ^9^ Importantly, when IDWG and FO were analyzed in the same data set, patients with lower IDWG had higher mortality throughout all FO ranges.^10^ The prevalence of FO post- and predialysis is alarmingly high as follows: >40% in previous data sets, small^11^ and large.^8^ The initial question, “how much fluid to remove,” therefore, portends more than merely practical relevance, as target weight likely needs to be corrected in almost every other patient.

Fluid restrictions seem indispensable in routine HD care, and high IDWG has traditionally been viewed as a measure of nonadherence.^12^ This concept is problematic, especially for patients who are dehydrated postdialysis, because target weight may have been set too low. The adherence aspect of IDWG has been described in multiple studies,^12^, ^13^, ^14^, ^15^ but without elaborating on the chronic overhydration issue. Several studies have been testing patient understanding^14^^,^^16^^,^^17^ and quality of life^14^^,^^18^ in patients receiving HD, but to our best knowledge, only 1 qualitative study^19^ has addressed aspects of fluid removal entirely from the patient’s side.

Following the analysis of Keane et al,^20^ our study aimed to explore patient perspectives and comprehension of target weight management and FO in HD, using semistructured interviews. Subsequently, a patient survey was derived from the interview results and dispensed to a larger HD cohort.

## Methods

### Study Design

This study represents a substudy of a larger qualitative study on the broad spectrum of personal experiences with HD. The original study’s scope addressed the following research fields: patients’ attitudes toward dialysis and their personal experiences, fluid management, blood pressure, and dietary practices. This substudy focuses specifically on fluid management and aims to provide an indepth qualitative analysis on the topic of FO and dry weight. Patients who participated in the semistructured interviews were already included in the nonrandomized, noninterventional exploratory study, “Closing the Loop in Hemodialysis: A Precision Medicine Approach—Part B (Establishing an Exploratory Dialysis Data-Pool).” In this study, we conducted absolute blood volume measurements using a dialysate bolus method,^21^^,^^22^ FO measurements using BIS,^23^^,^^24^ and pulse pressure wave analysis^25^ in maintenance HD patients over up to 14 subsequent HD sessions to interpret intradialytic blood volume dynamics and fluid status in a longitudinal fashion. The study and interview guide were approved by the ethics committee of the Medical University of Vienna (EK#: 2057/2020). This qualitative study was conducted in accordance with the Consolidated Criteria for Reporting Qualitative Research.^26^

### Interview Guide

Before data collection, an interview guide (Table S1) was prepared together with clinical nephrologists. For this analysis, the topics of interests were divided into the following: (1) fluid balance, FO, and drinking behavior, (2) dry-weight and weight documentation, and (3) dietary restrictions, containing open, closed, and prompt questions.

### Data Collection

The interviews were performed by local nephrologists and medical students at the chronic HD ward of Vienna General Hospital. All interviewers involved were previously trained by the authors of the interview guide under the lead of author UK. Patients were informed about the length and aim of the interviews and had to give their written and verbal consent. The interviews took place during an HD treatment session between November and December 2021. The interviews were pseudonymized after recording and transcription was outsourced to a local company (www.sprachdschungel.com/).

### Data Analysis

Interview transcripts were read carefully by the authors VR and JK with different levels of training and research backgrounds and were coded according to the principles of grounded theory.^27^ Meaningful quotes were identified through an open, axial, and selective coding process. Coding was discussed, compared, and subsequently pooled into main themes and subthemes. Repeated review sessions were performed to condense and redefine the generated themes to the last summarizing factor, ensuring conceptual strength. To provide a triangulation setting, nephrologists, the interview team, and the editors of the coding formed a focus group to discuss the findings. All interpretations were reevaluated and adjusted in a peer review model by discussing the themes and subthemes in 3 meeting sessions of 4 hours each with a total of 7 participants.

### Patient Survey

To objectify and quantify the patients’ agreement with the results of the qualitative analysis, a survey was designed based on the results of the interviews. The questions were developed together with clinical nephrologists and were designed to map onto the themes and subthemes. The survey was distributed in paper form during the routine HD session in the following 3 different HD centers: (1) Vienna General Hospital, (2) Vienna Dialysis Center, and (3) the HD ward of the Clinic Favoriten, Vienna. A total of 49 questions were worked out in German language containing categorial and Likert scale response formats (Table S2). All participants were asked for their consent, informed about the length of the survey, and asked for feedback if any obscurities arose while completing the questionnaire. The results were analyzed descriptively by indicating the distribution of responses in absolute numbers and percentages. Analysis of the patient survey was approved by the ethics committee of the Medical University of Vienna (EK#: 1434/2023).

## Results

### Demographics and Patient Characteristics

Among the 29 participants of our exploratory study, 25 agreed to the interview (20 self-identified male and 5 female participants). The median age of the interviewees was 66.0 years (interquartile range, 47.3-77.8). The median HD vintage was 1.9 years (interquartile range, 0.9-2.9), and BIS-detected overhydration of ≥15% extracellular water predialysis was present in 11 individuals (44%; Table 1).

**Table 1:** Patient Characteristics of the Interviewed Cohort at Baseline.

Baseline Characteristics	N (%)
Male	20 (80)
Female	5 (20)
Age (y)	66.0 (47.3-77.8)
Dialysis vintage (y)	1.9 (0.9-2.9)
BMI (kg/m^2^)	26.9 (22.9-30.3)
Access-type arteriovenous graft	12 (48)
Access-type catheter	13 (52)
Chronic heart failure	4 (16)
Arterial hypertension	22 (88)
Coronary artery disease	14 (56)
Diabetes mellitus	10 (40)
Cerebrovascular disease	5 (20)
Peripheral artery disease	5 (20)
Cancer	4 (16)
Lunge disease	4 (16)
Depression	2 (8)
Current smokers	6 (24)
BIS-defined overhydration predialysis^a^ (L)	2.8 (1.8-3.8)
BIS-defined relative overhydration predialysis^a^ (%)	13.6 (10.0-20.0)
BIS-defined relative overhydration > 15% ECW predialysis^a^	11 (44)
BIS-defined LTI difference to reference predialysis (kg/m^2^)	-0.2 (-2.6 to 2.6)
BIS-defined FTI difference to reference predialysis (kg/m^2^)	6.5 (4.5 to 11.3)
BIS-defined E/I predialysis	1.1 (0.9-1.2)
BIS-defined relative LTM predialysis (%)	44.8 (36.7-56.6)
BIS-defined relative fat predialysis (%)	37.9 (27.3-41.4)
Interview length (min)	41.5 (33.5-47.0)
PHQ-2 questionnaire score < 3	8/12 (32)
PHQ-2 questionnaire score ≥ 3	4/12 (16)
PHQ-2 questionnaire score^b^	1.04 ± 1.33

*Note:* continuous variables are presented as medians (IQRs); binary variables are presented in absolute numbers (%).

Abbreviations: BIS, bioimpedance spectroscopy; BMI, body mass index; ECW, extracellular water; E/I, extracellular water to intracellular water ratio; FTI, fat tissue index; IQR, interquartile range; LTI, lean tissue index; LTM, lean tissue mass; PHQ, Patient Health Questionnaire; SD, standard deviation.

a Data are presented as overall median (IQR) measurements over time.

b Data are presented as means (SD).

A total of 509 patients from 3 HD centers were approached for the survey and 148 (29.1%) agreed to participate. Of the 148 surveyed HD patients, 93 (62.8%) self-identified as men, 54 (36.5%) as women, and 1 (0.7%) as nonbinary. The median age was 66.0 years (interquartile range, 58.0-76.0). Fifty-one (34.7%) patients had a dialysis vintage of <1 year, 64 (43.5%) had 2-5 years, and 32 (21.8%) had ≥6 years. Most patients underwent 3 HD sessions/wk (137; 92.6%) and were not previously kidney transplanted (132; 89.8%; Table 2).

**Table 2:** Patient Characteristics at Baseline of the HD Cohort Who Underwent the Patient Survey (n = 148).

Baseline Characteristics	N (%)
Male	93 (62.8)
Female	54 (36.5)
Nonbinary	1 (0.7)
Age (y)	66.0 (58.0-76.0)
Dialysis vintage < 1 y	51 (34.7)
Dialysis vintage 2-5 y	64 (43.5)
Dialysis vintage 6-10 y	20 (13.6)
Dialysis vintage > 10 y	12 (8.2)
Transplanted kidney	15 (10.2)
No transplant kidney	132 (89.8)
None or <100 mL urine volume/d	51 (35.2)
100-500 mL urine volume/d	36 (24.8)
500-1,000 mL urine volume/d	32 (22.1)
>1,000 mL urine volume/d	26 (17.6)
3 dialysis sessions/wk	137 (92.6)
2 dialysis sessions/wk	10 (6.8)
1 dialysis sessions/wk	1 (0.7)

*Note*: continuous variables are presented as medians (IQRs); categorical variables are presented in absolute numbers (%).

Abbreviations: HD, hemodialysis; IQR, interquartile range.

### Qualitative and Quantitative Results

**Figure 1:**
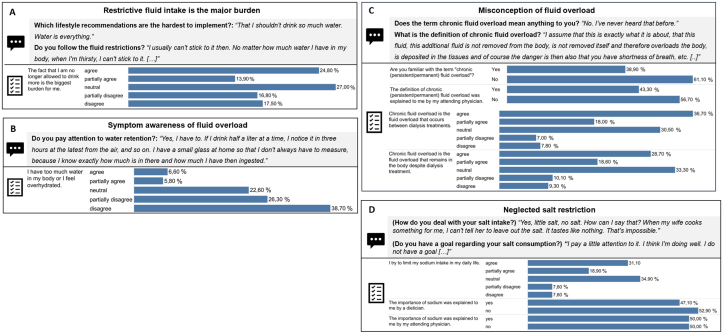
Qualitative quotes from the interviews and quantitative results compared for subthemes: (A) Restrictive fluid intake is the major burden. (B) Symptom awareness of fluid overload. (C) Misconception of fluid overload. (D) Neglected salt restriction of theme 1, “Being aware of fluid restrictions.”

**Figure 2:**
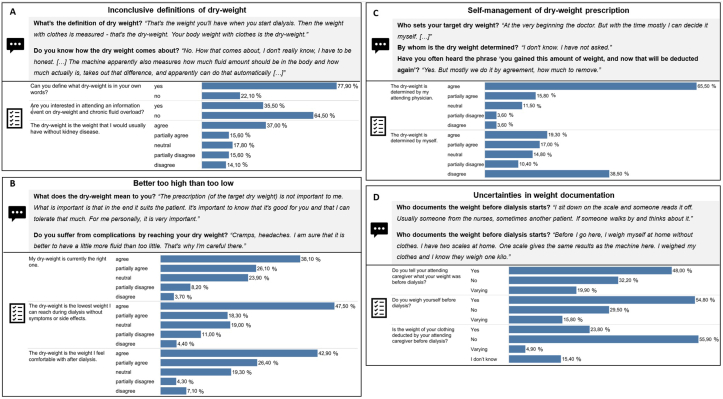
Qualitative quotes from the interviews and quantitative results compared for subthemes: (A) Inconclusive definitions of dry weight. (B) Better to high than too low. (C) Patient self-management of dry-weight prescription. (D) Uncertainties in weight documentation of theme 2, “Dry weight is a feel-good factor.”

**Figure 3:**
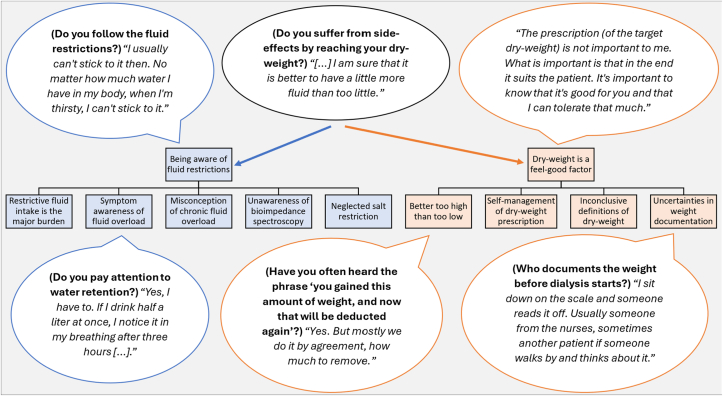
Key quotations of theme 1, “Being aware of fluid restrictions,” and theme 2, “Dry weight is a feel-good factor.”

**Figure 4:**
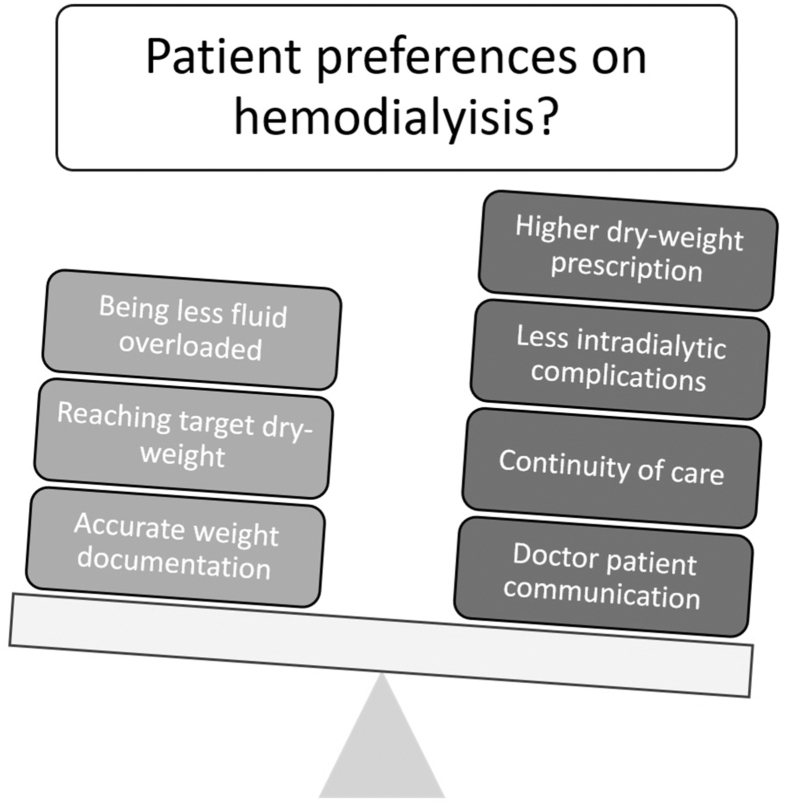
Elaborated preferences of the interviewed hemodialysis patients.

Two themes relevant to the topic, with 4-5 subthemes each, were identified (Table S3). Theme 1 represents the topic of FO (Fig 1), and theme 2 illustrates dry weight (Fig 2). Meaningful quotes are summarized in Fig 3. A profile for preferences on HD is elaborated in Fig 4. All quotations can be obtained from Table S3.

#### Theme 1: Being Aware of Fluid Restrictions

Drinking restrictions seemed omnipresent in daily life and difficult to comply with, as reflected in subtheme 1, “Restrictive fluid intake is the major burden” (Fig 1A**)**. FO was deemed as asymptomatic and was frequently accepted. In the survey, however, only 24.8% (n = 34) fully agreed to subtheme 1, namely that “no longer (being) allowed to drink … is the biggest burden” (question no. 48d; Table S2).

In subtheme 2, “Symptom awareness of FO” (Fig 1B), patients mentioned dyspnea and edema as the predominant symptoms that usually occurred quickly, increasing pressure to adhere to drinking restrictions. Eighty-nine (65.0%) surveyed HD patients, however, did not feel overhydrated, suggesting limited symptom awareness (question no. 48c; Table S2).

In subtheme 3, “Misconception of chronic FO” (Fig 1C), chronic FO was confused with symptomatic IDWG. FO as a term was often unknown and was never explained before, as confirmed by the survey (61.1% and 56.7%, respectively; question nos. 22 and 25; Table S2). In all, 54.7% fully/partially agreed that chronic FO occurs between dialysis treatments, indicating the confusion with IDWG (question no. 27a; Table S2).

In subtheme 4, “Unawareness of bioimpedance spectroscopy,” BIS could partly be assigned to FO. Few patients showed technical understanding, but the term was rather unfamiliar or confusing. Patients requested more information on BIS.

In subtheme 5, “Neglected salt restriction” (Fig 1D), patients frequently delegated dietary responsibility to relatives (including partner or spouse), who managed their salt intake. In the survey, sodium restriction was followed by 50% and the importance of sodium had been explained by 50% of the doctors (question nos. 46a and 34; Table S2).

#### Theme 2: Dry Weight Is a Feel-Good Factor

Patients demonstrated having “Inconclusive definitions of dry weight” (subtheme 1; Fig 2A), which involved a rough association with proper blood pressure or a fluid-depleted state. Other definitions were described as being misleading. In all, 77.9% of surveyed patients reported being able to define dry weight, whereas only 37.0% fully agreed that dry weight is the weight that patients would usually have without kidney disease (question nos. 13 and 18e; Table S2). In all, 64.5% were not interested in an educational event on dry weight and FO (question no. 49; Table S2).

In subtheme 2, “Better too high than too low” (Fig 2B), many patients clearly stated that they preferred a higher dry weight—regardless of BIS and doctor’s prescription—to avoid unpleasant intradialytic complications and feel comfortable postdialysis. In the survey, patients associated dry weight strongly with the lowest reachable weight without intradialytic side effects and the weight at which they felt comfortable after dialysis (65.8% and 69.3% partial/full agreement, respectively; question nos. 18f and 18a; Table S2).

In subtheme 3, “Self-management of dry-weight prescription” (Fig 2C), dry weight was classified as “negotiable.” Patients emphasized autonomy, apart from medical recommendations. However, most surveyed patients fully/partially agreed that their current dry weight was the right one and was prescribed by their doctors (64.2% and 81.3%, respectively; question nos. 48a and 18c; Table S2).

In subtheme 4, “Uncertainties in weight documentation” (Fig 2D), patients reported that it was usually different by whom the body mass was documented from the scales predialysis. It was confusing whether the weight was calculated with or without clothing. Survey responses did not show a consistent trend (question nos. 20, 20a, and 21; Table S2), indicating a general lack of clarity.

## Discussion

We have demonstrated that patients treated with HD feel a clear burden from management of fluid status but have significant knowledge gaps about FO, have a lack of interest in engaging with education, and rarely adhere to salt and fluid restrictions. These findings are particularly stark, given the well-described associations between management of FO and both acute symptoms during HD and long-term outcomes.^2^^,^^7^^,^^8^

Fluid status varies in patients with HD over each week, with regular defined periods of fluid accumulation and removal, which can distract from gross, underlying volume expansion.^5^ In one of our HD centers participating in the survey, predialysis FO was prevalent in 41.6% of patients. Patients frequently confounded chronic FO with symptoms of IDWG, exemplified by numerous patients who considered chronic FO to occur between dialysis rather than to persist after dialysis. The majority of patients also indicated that their dry weight was correct and that they did not feel fluid overloaded. This confusion illustrates a significant gap in knowledge, which exemplifies the issue of FO being the “elephant in the room that no one sees (and feels).”^28^

In our interview cohort, dry weight was mostly described as the weight after fluid removal, but in some instances, the term seemed to have been misinterpreted. The survey suggested that the vast majority of patients were confident that they would be able to define dry weight (77.9%), but we do not know if this definition would have been sensible or if dry weight might have been confounded, similar to what we have learned from our interview cohort. Confusion regarding the terminology, paired with imprecise calculations of predialysis body mass (with/without clothing and with/without deduction of clothing mass), highlights the need for standardized measurement methods of weight and fluid status in daily routine. There was also confusion among interviewees as to who exactly determined their dry weight, although patients generally preferred to set dry weight based on consensus. Interestingly, 81.3% of surveyed patients fully/partially agreed that their dry weight was prescribed by their physician and not by themselves. This practice, however, may have been center specific. A study on stage 5 of chronic kidney disease^29^ demonstrated that most patients preferred to leave decisions entirely to health care professionals, whereas data by Keane et al^20^ revealed that the majority of HD patients were given the final say in deciding how much fluid to remove. Our results, however, suggested that only a minority of patients want to be actively involved in fluid management.

In our interview study, fluid restriction was identified as the major burden in HD. For patients who were unable to adhere to fluid restrictions, drinking was associated with a sense of guilt, compounded by the rapid onset of FO-related symptoms, triggering anxiety about fluid intake and causing profound psychological and physical distress. A cofactor to be mentioned in this context was the tendency to neglect sodium restriction with a low interest in nutritional advice in general, demonstrated both in interviews and our survey. According to a systematic review, fluid restriction is a multifaceted burden including social interference with role models, being overwhelmed by too much information, negotiating personal priorities, or simply withstanding physiological needs.^15^ In a previous study, patients bothered by fluid restriction were willing to extend HD treatment time if they were allowed unrestricted fluid intake in return, but symptoms related to FO were not significantly linked to their willingness to adopt volume mitigation strategies.^30^ Thus, it could be assumed that fluid restriction is perceived as more stressful than symptom burden of FO, which is consistent with our qualitative findings that FO-related symptoms were better tolerated.

Interestingly, the importance and burden of fluid restriction was less clearly emphasized in the survey responses. This divergence may reflect social desirability bias, possibly contributed by the difference in methodologies between interview and survey formats. The interview setting may have facilitated a greater sense of being understood among patients, thereby promoting more focused and open communication of their burden. However, the observed discrepancy may also stem from the small sample size in combination with the highly individualized interpretation and implementation of fluid restriction in the context of HD.

There was also a clear theme linked to the burden from intradialytic symptoms and complications linked to fluid removal. Patients gave priority to prescribe dry weight in such a way that intradialytic complications such as cramps or dizziness would be avoided. Surveyed patients tended to agree that dry weight was the weight they felt comfortable with postdialysis rather than the weight they would have without kidney disease (69.3% vs 52.6%). Concordantly, a qualitative analysis by Glyde et al^19^ previously defined the theme “Volume of Fluid to be removed,” in which they referred to fluid removal strategies in which patients strictly avoided short-term complications on receiving HD in advance, with most unaware of the long-term complications caused by excessive or insufficient ultrafiltration volumes. Further results from another study showed that 30% of HD patients would easily tolerate postdialysis FO in favor of ending their dialysis session earlier, whereas 56% considered flexibility in their diet and fluid restrictions to be more important than reaching their target weight.^20^

Taken together, these results have clinically relevant implications. The burden on patients is clear, but formal “education” does not appear to be desirable. Instead, future strategies should integrate personalized, dialog-based communication and behavioral support models to increase patient engagement. But how can we support better informed patient involvement in the management of fluid during HD? BIS offers one possibility for regular feedback to patients, as regular information on current fluid status can increase awareness of the appropriate dry weight. Monitoring details of the disease has been shown to give patients a sense of control and a feeling of being better informed about the disease progression and treatment.^31^ Koch-Weser et al^32^ noted that many education programs overlook core principles of health literacy and fail to address key psychosocial and prognostic concerns. Effective communication in dialysis care relies on tailored, culturally sensitive strategies and a strong focus on health literacy. Studies have shown that individualized dietary counseling and improved interdisciplinary coordination enhance patient understanding,^33^ whereas bidirectional dialog (ie, an open exchange in which patients are encouraged to ask questions, express preferences, and participate in care decisions) and clearly defined care roles help overcome common communication barriers.^34^ For older patients, clear and preference-based communication is essential for informed decision-making.^35^

## Limitations

When applying grounded theory, there is a methodological risk of subjective interpretation because there is no quantitative verification of the results. To maintain objectivity and ensure the external validity of the developed theories, the results were repeatedly peer reviewed in the triangulation setting, which included students as well as nephrologists experienced in HD care. The small number of interviews and the unequal gender distribution are limitations and could have led to a gender-specific bias. A previous qualitative analysis demonstrated that gender disparities in chronic kidney disease are shaped by social roles and expectations. Women often reported feeling emotionally vulnerable and were more actively involved in managing their health, whereas men tended to downplay their illness and rely more on others.^36^ Twenty out of 25 interview participants in our study identified as male may have influenced the thematic focus of our findings, potentially underrepresenting perspectives characterized by emotional burden, proactive help seeking, or openness to educational support. The gender distribution and age structure in the survey, on the other hand, were almost identical.

Furthermore, there was the possibility of selection bias in the small interview cohort, which was recruited in only 1 HD center. Our study was conducted in a single country (Austria) and limited to German-speaking patients. Cultural norms around autonomy, health communication, and physician trust can vary significantly across health care systems. In German countries, nephrology care is highly physician centered and treatment decisions are often led by medical professionals,^37^ which may explain why most patients in our cohort expected dry weight to be prescribed by their nephrologist. These cultural characteristics may limit generalizability to countries with more shared decision-making practices.

This survey was not validated externally and must be viewed with all the associated limitations. The response rate of 29.1% introduces potential selection bias, especially given that language and literacy barriers may have excluded patients with different sociocultural backgrounds. The survey was administered in German only, which likely restricted participation by nonnative speakers. The analysis used descriptive statistics without adjustment for confounders. Although appropriate for an exploratory study, the lack of inferential analysis limits our ability to interpret associations or draw causal inferences.

Although most of the quantitative results were consistent with the themes and subthemes identified in the interview study, discrepancies in some findings may partially question its validity, which would need to be better confirmed in subsequent studies.

## Conclusions

The preference for symptom avoidance over long-term volume control appears to be a dominant behavior, which raises a clinical challenge. Our analyses revealed not only significant knowledge gaps regarding chronic FO and dry weight but also a lack of interest, with almost two-thirds of participants expressing no interest in attending an educational event on fluid-related topics. Innovative, patient-centered, and interdisciplinary communication strategies are required to involve individuals more closely into fluid management, thereby preventing the long-term consequences of chronic FO and to minimize the clinical burden of IDWG.
