# Supplementary material for: The Dry-Weight Dilemma: Survey Results Based on a Qualitative Interview Study on Fluid Overload in Hemodialysis
Source: Kidney Med. 2026 Mar 10;8(5):101320. doi: 10.1016/j.xkme.2026.101320 (PMC13091120; doi:10.1016/j.xkme.2026.101320)
Supplement: Supplementary File (PDF) — Tables S1-S3. [file mmc1.pdf]

Supplementary Material

Table S1: Interview guide containing potential open, closed and prompt questions.

Q#	Question
	**Fluid balance, fluid overload and drinking behaviour**
1)	Do you actually know what methods there are to determine whether you have too much or too little water in your body?
2)	Are you familiar with the BVM (Blood Volume Monitoring) curve on the dialysis machine? Can you tell us something about it?
3)	Body Composition Monitor measurements were carried out each time using a bioimpedance device. Do you know what is measured there and why?
4)	Do you have edema, or do you know what it is?
5)	Do you suffer from dyspnea?
6)	What physical symptoms of fluid in your body do you know?
7)	Do you actually have a specific drinking amount that you stick to?
8)	Do you regularly monitor your body for water retention?
9)	Are you worried about water retention?
10)	Can you resist a strong feeling of thirst? Do you often drink too much even though you were told to drink less?
11)	Do you feel unwell after drinking too much in a day?
12)	Have you been told to drink less or more? Can you tell me details?
13)	How much of a burden is it for you to only be allowed to drink a certain amount according to medical recommendations?
	**Dry-weight and weight documentation**
14)	What do you understand by the term dry-weight?
15)	How and by whom is your dry weight determined?
16)	Do you have a feeling for which dry-weight is good for you?
17)	… Can you give me a number (dry-weight)?
18)	Do you think your current dry-weight is the right one?
19)	Has it ever happened that your dry-weight was set incorrectly?
20)	Are you worried that the dry-weight is set too low?
21)	In this context, do you fear possible undesirable side effects (cramps, drops in blood pressure, etc.)?
22)	How important do you think dry-weight is compared to other things that need to be considered during dialysis?
23)	When you come for dialysis, are you actually always weighed, on the scale? Why is this actually being done?
24)	And who does the weighing or who writes down the weight? Do you remember your weight or does your attending nurse do that?
25)	Do you know the sentence, "you have gained so much weight, we will now take this amount off again?"
26)	All right, now we've discussed gaining weight, but now I want to know something else from you: How do you think gaining weight relates to your dry-weight?
27)	Do you know what interdialytic weight gain is?
28)	What exactly is it that you gain between dialysis sessions?
29)	Are you worried about changes of your weight?

30)	Do you feel ashamed if you think the interdialytic weight gain is perceived as too high by your healthcare team?
31)	What are your biggest obstacles to achieve your daily goals regarding weight and fluid intake?
	**Dietary aspects**
32)	Please tell me something about your drinking and eating habits (wait and see what comes from the patient).
33)	Do you have a goal regarding your salt intake?
34)	How do you feel about the advice from nurses, dietitians and doctors?
35)	Can you tell me details about the dietary advices you have received?
36)	Have you been told to consume less or more salt? Do you know what salt is contained in?
37)	Are you burdened by strict rules about what you should and should not eat between dialysis sessions?
38)	Do you adhere to these rules in daily life?
39)	Were there any moments when you were shamed or reprimanded by healthcare workers for your eating and drinking habits between dialysis sessions?

Table S2: Patient survey on the topics of dry-weight and chronic fluid overload.

Q#	Question	Answer	N (%)
**1)**	**Age (median [IQR])**		
			66.0 (58.0-76.0)
**2)**	**Sex**		
		Male	93 (62.8%)
		Female	54 (36.5%)
		Non-binary	1 (0.7%)
		Missing data	0
**3)**	**How long have you been a dialysis patient?**		
		< 1 year	51 (34.7%)
		2-5 years	64 (43.5%)
		6-10 years	20 (13.6%)
		> 10 years	12 (8.2%)
		Missing data	1
**4)**	**Do you have a transplanted kidney?**		
		Yes	15 (10.2 %)
		No	132 (89.8%)
		Missing data	1
**5)**	**How much urine do you produce per day?**		
		None or < 100ml	51 (35.2%)
		100-500 ml	36 (24.8%)
		500-1000 ml	32 (22.1%)
		> 1000ml	26 (17.9%)
		Missing data	3
**6)**	**How many dialysis sessions do you have per week?**		
		3	137 (92.6%)
		2	10 (6.8%)
		1	1 (0.7%)

		Missing data	0
**7)**	**Do you know the cause of your kidney failure or kidney disease?**		
		Yes	85 (57.4%)
		No	63 (42.6%)
		Missing Data	0
**7)a)**	**If you checked “No” for question 7): What is the reason?**		
		The cause of the kidney failure could not be determined	24 (40.7%)
		I don’t know	35 (59.3%)
		Invalid answer	4
		Missing data	4
**8)**	**Is the following statement correct: I believe my attending caregiver knows the cause of my kidney failure (kidney disease).**		
		Yes	38 (27.3%)
		No	38 (27.3%
		I don’t know	63 (45.3%)
		Missing data	9
**9)**	**Is the following statement correct: I believe my attending physician knows the cause of my kidney failure (kidney disease).**		
		Yes	81 (57.5%)
		No	22 (15.6%)
		I don’t know	38 (27.0%)
		Missing data	7
**10)**	**Is the following statement correct: The cause of my kidney failure (kidney disease) was explained to me by my attending physician.**		
		Yes	80 (57.6%)
		No	59 (42.5%)
		Missing data	9
**11)**	**Is the following statement correct: The cause of my kidney failure (my kidney disease) was explained to me by my attending caregiver.**		

		Yes	35 (25.9%)
		No	100 (74.1%)
		Missing data	13
**12)**	**Are you familiar with the term ‘dry-weight’?**		
		Yes	124 (84.9%)
		No	22 (15.1%)
		Missing data	2
**13)**	**Can you define what dry-weight is in your own words?**		
		Yes	113 (77.9%)
		No	32 (22.1%)
		Missing data	3
**14)**	**Is the following statement correct: I believe my attending caregiver knows the definition of dry-weight.**		
		Yes	120 (83.9%)
		No	7 (4.9%)
		I don’t know	16 (11.2%)
		Missing data	5
**15)**	**Is the following statement correct: I think my attending physician knows the definition of dry-weight.**		
		Yes	133 (92.4%)
		No	3 (2.1%)
		I don’t know	8 (5.6%)
		Missing data	4
**16)**	**Is the following statement correct: The definition of dry-weight was explained to me by my attending physician.**		
		Yes	101 (72.1%)
		No	39 (27.9%)
		Missing data	8
**17)**	**Is the following statement correct: The definition of dry-weight was explained to me by my attending caregiver.**		
		Yes	90 (64.3%)

		No	50 (35.7%)
		Missing data	8
	**Mark your answer in the appropriate box using the scale:**		
**18)a)**	**The dry-weight is the weight I feel comfortable with after dialysis.**		
		Agree	60 (42.9%)
		Partially agree	37 (26.4%)
		Neutral	27 (19.3%)
		Partially disagree	6 (4.3%)
		Disagree	10 (7.1%)
		Missing data	8
**18)b)**	**The dry-weight is determined by my attending caregiver.**		
		Agree	50 (36.0%)
		Partially agree	23 (16.6%)
		Neutral	22 (15.8%)
		Partially disagree	9 (6.5%)
		Disagree	35 (25.2%)
		Missing data	9
**18)c)**	**The dry-weight is determined by my attending physician.**		
		Agree	91 (65.5%)
		Partially agree	22 (15.8%)
		Neutral	16 (11.5%)
		Partially disagree	5 (3.6%)
		Disagree	5 (3.6%)
		Missing data	9
**18)d)**	**The dry-weight is determined by myself.**		
		Agree	26 (19.3%)
		Partially agree	23 (17.0%)
		Neutral	20 (14.8%)
		Partially disagree	14 (10.4%)
		Disagree	52 (38.5%)
		Missing data	13

**18)e)**	**The dry-weight is the weight that I would usually have without kidney disease.**		
		Agree	50 (37.0%)
		Partially agree	21 (15.6%)
		Neutral	24 (17.8%)
		Partially disagree	21 (15.6%)
		Disagree	19 (14.1%)
		Missing data	13
**18)f)**	**The dry-weight is the lowest weight I can reach during dialysis without symptoms or side effects.**		
		Agree	65 (47.5%)
		Partially agree	25 (18.3%)
		Neutral	26 (19.0%)
		Partially disagree	15 (11.0%)
		Disagree	6 (4.4%)
		Missing data	11
**18)g)**	**The dry-weight is a fixed value and usually does not change.**		
		Agree	16 (11.9%)
		Partially agree	17 (12.6%)
		Neutral	27 (20.0%)
		Partially disagree	23 (17.0%)
		Disagree	52 (38.5%)
		Missing data	13
**18)h)**	**The dry-weight is a variable value and can often change.**		
		Agree	60 (43.8%)
		Partially agree	39 (28.5%)
		Neutral	27 (19.7%)
		Partially disagree	6 (4.4%)
		Disagree	5 (3.7%)
		Missing data	11
**19)**	**Do you weigh yourself before dialysis?**		

		Yes	80 (54.8%)
		No	43 (29.5%)
		Varying	23 (15.8%)
		Missing data	2
**20)**	**Do you tell your attending caregiver what your weight was before dialysis?**		
		Yes	70 (48.0%)
		No	47 (32.2%)
		Varying	29 (19.9%)
		Missing data	2
**20)a)**	**If you have ticked "Yes" or "Varying" for question 20): Is the weight of your clothing deducted by your attending caregiver before dialysis?**		
		Yes	35 (36.8%)
		No	55 (57.9%)
		Varying	5 (5.3%)
		Invalid answer	22
		Missing data	4
**21)**	**Is the weight of your clothing deducted by your attending caregiver before dialysis?**		
		Yes	34 (23.8%)
		No	80 (55.9%)
		Varying	7 (4.9%)
		I don’t know	22 (15.4%)
		Missing data	5
**22)**	**Are you familiar with the term "chronic (persistent/permanent) fluid overload"?**		
		Yes	56 (38.9%)
		No	88 (61.1%)
		Missing data	4
**23)**	**Do you think you could define in your own words what chronic (persistent/permanent) fluid overload is?**		
		Yes	57 (39.6%)

		No	87 (60.4%)
		Missing data	4
**24)**	**Is the following statement correct: I believe my attending caregiver knows the definition of chronic (persistent/permanent) fluid overload.**		
		Yes	92 (64.8%)
		No	4 (2.8%)
		I don’t know	46 (32.4%)
		Missing data	6
**25)**	**Is the following statement correct: I believe my attending physician knows the definition of chronic (persistent/permanent) fluid overload.**		
		Yes	121 (83.5%)
		No	1 (0.7%)
		I don’t know	23 (15.9%)
		Missing data	3
**25)**	**The definition of chronic (persistent/permanent) fluid overload was explained to me by my attending physician.**		
		Yes	61 (43.3%)
		No	80 (56.7%)
		Missing data	7
**26)**	**The definition of chronic (persistent/permanent) fluid overload was explained to me by my attending caregiver.**		
		Yes	47 (33.3%)
		No	94 (66.7%)
		Missing data	7
	Mark your answer in the appropriate box using the scale:		
**27)a)**	**Chronic fluid overload is the fluid overload that occurs between dialysis treatments.**		
		Agree	47 (36.7%)
		Partially agree	23 (18.0%)
		Neutral	39 (30.5%)
		Partially disagree	9 (7.0%)

		Disagree	10 (7.8%)
		Missing data	20
**27)b)**	**Chronic fluid overload is the fluid overload that remains in the body despite dialysis treatment.**		
		Agree	37 (28.7%)
		Partially agree	24 (18.6%)
		Neutral	43 (33.3%)
		Partially disagree	13 (10.1%)
		Disagree	12 (9.3%)
		Missing data	19
**28)**	**Is the following statement correct: I believe that my attending physician is aware of the influence of dialysis, including dry-weight, on blood pressure.**		
		Yes	122 (86.5%)
		No	2 (1.4%)
		I don't know	17 (12.1%)
		Missing data	7
**29)**	**Is the following statement correct: I believe that my attending caregiver is aware of the influence of dialysis, including dry-weight, on blood pressure.**		
		Yes	104 (74.3%)
		No	6 (4.3%)
		I don't know	30 (21.4%)
		Missing data	8
**30)**	**Is the following statement correct: The influence of dialysis, including dry-weight, on blood pressure was explained to me by my attending physician.**		
		Yes	95 (68.4%)
		No	44 (31.7%)
		Missing data	9
**31)**	**Is the following statement correct: The influence of dialysis, including dry-weight, on blood pressure was explained to me by my attending caregiver.**		
		Yes	79 (58.5%)
		No	56 (41.5%)

		Missing data	13
	**Mark your answer in the appropriate box using the scale:**		
**32)a)**	**The blood pressure is usually too high if the dry-weight is too high.**		
		Agree	33 (24.3%)
		Partially agree	25 (18.4%)
		Neutral	49 (36.0%)
		Partially disagree	15 (11.0%)
		Disagree	14 (10.3%)
		Missing data	12
**32)b)**	**Blood pressure falls during dialysis because water is removed.**		
		Agree	60 (43.8%)
		Partially agree	35 (25.6%)
		Neutral	24 (17.5%)
		Partially disagree	15 (11.0%)
		Disagree	3 (2.2%)
		Missing data	11
**32)c)**	**The dry-weight can still be too high even if the blood pressure is low.**		
		Agree	28 (21.2%)
		Partially agree	32 (24.2%)
		Neutral	56 (42.4%)
		Partially disagree	8 (6.1%)
		Disagree	8 (6.1%)
		Missing data	16
**32)d)**	**If the blood pressure is still too low before dialysis, the dry-weight must be better adjusted.**		
		Agree	29 (22.7%)
		Partially agree	21 (16.4%)
		Neutral	68 (53.1%)
		Partially disagree	5 (3.9%)
		Disagree	5 (3.9%)
		Missing data	20

**33)**	**Do you know what sodium is contained in?**		
		Yes	92 (66.7%)
		No	46 (33.3%)
		Missing data	10
**34)**	**Is the following statement correct: The importance of sodium was explained to me by my attending physician.**		
		Yes	69 (50.0%)
		No	69 (50.0%)
		Missing data	10
**35)**	**Is the following statement correct: The importance of sodium was explained to me by my attending caregiver.**		
		Yes	58 (42.3%)
		No	79 (57.7%)
		Missing data	11
**36)**	**Is the following statement correct: The importance of sodium was explained to me by a dietician.**		
		Yes	64 (47.1%)
		No	72 (52.9%)
		Missing data	12
**37)**	**Do you know what potassium is contained in?**		
		Yes	88 (62.9%)
		No	11 (7.9%)
		Partially	41 (29.3%)
		Missing data	8
**38)**	**Is the following statement correct: The importance of potassium was explained to me by my attending physician.**		
		Yes	104 (75.4%I
		No	34 (24.6%)
		Missing data	10
**39)**	**Is the following statement correct: The importance of potassium was explained to me by my attending caregiver.**		

		Yes	83 (61.0%)
		No	53 (39.0%)
		Missing data	12
**40)**	**Is the following statement correct: The importance of potassium was explained to me by a dietician.**		
		Yes	93 (68.4%)
		No	43 (31.6%)
		Missing data	12
**41)**	**Do you know what phosphate is contained in?**		
		Yes	63 (45.3%)
		No	35 (25.2%)
		Partially	41 (29.5%)
		Missing data	9
**42)**	**Is the following statement correct: The importance of phosphate was explained to me by my attending physician.**		
		Yes	77 (55.8%)
		No	61 (44.2%)
		Missing data	10
**43)**	**Is the following statement correct: The importance of phosphate was explained to me by my attending caregiver.**		
		Yes	54 (39.7%)
		No	82 (60.3%)
		Missing data	12
**44)**	**Is the following statement correct: The importance of phosphate was explained to me by a dietician.**		
		Yes	83 (60.6%)
		No	54 (39.4%)
		Missing data	11
**45)**	**Have you ever received nutritional advice from a nutritionist?**		
		Yes	109 (79.6%)
		No	28 (20.4%)

		Missing data	11
	**<u>Mark your answer in the appropriate box using the scale:</u>**		
**46)a)**	**I try to limit my sodium intake in my daily life.**		
		Agree	41 (31.1%)
		Partially agree	25 (18.9%)
		Neutral	46 (34.9%)
		Partially disagree	10 (7.6%)
		Disagree	10 (7.6%)
		Missing data	16
**46)b)**	**I try to limit my potassium intake in my daily life.**		
		Agree	51 (37.8%)
		Partially agree	36 (26.7%)
		Neutral	35 (25.9%)
		Partially disagree	8 (5.9%)
		Disagree	5 (3.7%)
		Missing data	13
**46)c)**	**I try to limit my phosphate intake in my daily life.**		
		Agree	41 (30.8%)
		Partially agree	31 (23.3%)
		Neutral	39 (29.3%)
		Partially disagree	13 (9.8%)
		Disagree	9 (6.8%)
		Missing data	15
**47)**	**Dialysis is a necessary evil for me, but I accept it.**		
		Yes	118 (91.5%)
		No	11 (8.5%)
		Missing data	19
	**<u>If you answered "No" to question 47): What is the reason?</u>**		
**47)a)**	**Dialysis is better than described above.**	Yes	9 (81.8%)
		No	2 (18.2%)
		Invalid answer	65

		Missing data	0
**47)b)**	**Dialysis is worse than described above.**		
		Yes	1 (14.3%)
		No	6 (85.7%)
		Invalid answer	43
		Missing data	4
	**Mark your answer in the appropriate box using the scale:**		
**48)a)**	**My dry-weight is currently the right one.**	Agree	51 (38.1%)
		Partially agree	35 (26.1%)
		Neutral	32 (23.9%)
		Partially disagree	11 (8.2%)
		Disagree	5 (3.7%)
		Missing data	14
**48)b)**	**I would like to have a higher dry-weight in order to suffer fewer complications during dialysis.**		
		Agree	14 (10.5%)
		Partially agree	10 (7.5%)
		Neutral	28 (21.1%)
		Partially disagree	36 (27.1%)
		Disagree	45 (33.8%)
		Missing data	15
**48)c)**	**I have too much water in my body or I feel overhydrated.**		
		Agree	9 (6.6%)
		Partially agree	8 (5.8%)
		Neutral	31 (22.6%)
		Partially disagree	36 (26.3%)
		Disagree	53 (38.7%)
		Missing data	11
**48)d)**	**The fact that I am no longer allowed to drink more is the biggest burden for me.**		

		Agree	34 (24.8%)
		Partially agree	19 (13.9%)
		Neutral	37 (27.0%)
		Partially disagree	23 (16.8%)
		Disagree	24 (17.5%)
		Missing data	11
**49)**	**Are you interested in attending an information event on dry-weight and chronic fluid overload?**		
		Yes	49 (35.5%)
		No	89 (64.5%)
		Missing data	10

Table S3: Themes, Subthemes and topic related representative quotes.

Theme	Subtheme	Quote
**Being aware of fluid restrictions**		
	Restrictive fluid intake is the major burden	(Which lifestyle recommendations are the hardest to implement?) That I shouldn't drink so much water. Water is everything.<br><br>(Do you follow the fluid restrictions?) I usually can't stick to it then. No matter how much water I have in my body, when I'm thirsty, I can't stick to it. […]
	Symptom awareness of fluid overload	(Do you feel psychological pressure regarding fluid intake restrictions?) I immediately feel when I press under the ribs when I have too much liquid. I can feel it immediately with my finger. Then I don't even need to get on the scale. I know my weight. It all comes with time.<br><br>(Do you pay attention to water retention?) Yes, I have to. If I drink half a liter at once, I notice it in my breathing after three hours, and so on. I have a small glass at home so that I don't always have to measure, because I know exactly how much is inside there and how much I have then ingested.
	Misconception of chronic fluid overload	(Definition of chronic fluid overload) I assume that this is exactly what it is about, that this fluid, this additional fluid is not removed from the body, is not removed itself and therefore overloads the body, is deposited in the tissues and of course the danger is then also that you have shortness of breath, etc. That then goes to the lungs, I am already aware of that. I'm not a medical doctor, that's not my field.<br><br>(How does the chronic excess of fluid occur?) If the kidney does not process that into urine and excrete it. The kidney has a filtering function. If it doesn't do that, the creatinine is high and so is the fluid. If you drink a lot, there is accordingly a lot of fluid in circulation.<br><br>(Does the term chronic fluid overload mean anything to you?) No. I've never heard that before.
	Unawareness of	(Do you know what the Body Composition Monitor measures?) The conductivity of my body. I suppose the more the body is saturated with water, the better the conductivity. The drier, the weaker.(BCM)

	bioimpedance spectroscopy	(Do you know what the Body Composition Monitor measures?) No, no one said anything.(BCM).<br><br>(Do you know what the Body Composition Monitor measures?)<br>I think that's just this measurement of how much fluid gets away. (Why is this measurement performed?) Because you have to look at exactly how much fluid is in the body.
	Neglected salt restriction	(How do you deal with your salt intake?) Yes, little salt, no salt. How can I say that? When my wife cooks something for me, I can't tell her to leave out the salt. It tastes like nothing. That's impossible.<br><br>(Do you watch your salt intake?) No. I don't consume much, just normal. But you can't do without salt.<br><br>(Do you watch your salt intake?) Not really. I've always been a salty eater.<br><br>(Do you have a goal regarding salt consumption?) I pay a little attention to it. I think I'm doing well. I do not have a goal […]
**Dry-weight is a feel-good factor**		
	Inconclusive definitions of dry-weight	(What's the definition of dry-weight?) That's the weight you'll have when you start dialysis. Then the weight with clothes is measured - that's the dry-weight. Your body weight with clothes is the dry-weight.<br><br>(What's the definition of dry-weight?) That is the fluid where the limit is, where the blood pressure is halfway within the limits. Anything above that is the fluid that has to be taken off.<br><br>(Do you know how the dry-weight comes about?) No. How that comes about, I don't really know, I have to be honest. I put something together, whether it is correct is another question. The machine apparently also measures how much fluid amount should be in the body and how much actually is, takes out that difference and apparently can do that automatically, at least that's how I understood it, and that's then the dry-weight that's left over.
	Better too high than too low	(Do you suffer from side-effects by reaching your dry-weight?) Cramps, headaches. I am sure that it is better to have a little more fluid than too little. That's why I'm careful there.

		(What does the dry-weight mean to you?) The prescription (of the target dry-weight) is not important to me. What's important is that in the end it suits the patient. It's important to know that it's good for you and that I can tolerate so much. For me personally, it is very important.<br><br>(Were there any recent changes of your dry-weigth?) When it's too dry, it sometimes goes down to 56.8 kg. I feel worse than when it's at 57 kg. But sometimes doctors say that I still have liquids and that they can reduce them the weight to 56kg or 56.7kg but I start to feel very tired then. For me it's better when it's 57kg.<br><br>(Who sets your target dry-weight?) […] And also when I feel that I sometimes get cramps, then it is a sign that the dry-weight is set too low. Then I always say that I probably need half a liter more.<br><br>(What happens when the dry-weight is too low?) I start to feel very exhausted, I've got pain in my back, everywhere, it goes from my neck down the body. I feel very uncomfortable, I don't know how to sit or to lie and I start to feel very dizzy. Sometimes I even cannot stand because everything goes around moves and I feel that I… I forgot the word… That I can fall to the floor. And that I need to grab something or sit because I can't walk.<br><br>(Do you suffer from side-effects by reaching your dry-weight?) Reaching the dry-weight leads to cramps. For some time now I have increased cramps at home at night.
	Self-management of dry-weight prescription	(Who sets your target dry-weight?) At the very beginning the doctor. But with the time, mostly I can decide it myself. […]<br><br>(Have you often heard the phrase "you gained this amount of weight, and now that will be removed again?") Yes. But mostly we do it by agreement, how much to remove.<br><br>(By whom is the dry-weight determined?) I don't know. I have not asked.<br><br>(Have you often heard the phrase "you gained this amount of weight, and now that will be removed again?") No. I usually tell the nurses or the nurse practitioners how much weight I want to lose. But that always refers to the dry-weight.

	Uncertainties in weight documentation	(Who documents the weight before dialysis starts?)<br>I sit down on the scale and someone reads it off. Usually someone from the nurses, sometimes another patient. If someone walks by and thinks about it.<br><br>(Who documents the weight before dialysis starts?) I always write down the weight.<br><br>I'll keep it in mind.<br><br>The nurse writes that down.<br><br>I'm usually there early enough in the morning that I stand on it, announce the weight, and that's how it's entered into the machine.<br><br>Before I go here, I weigh myself at home without clothes. I have two scales at home. One scale gives the same results as the machine here. I weighed my clothes and I know they weigh one kilo.
